# Computational analysis of calculated physicochemical and ADMET properties of protein-protein interaction inhibitors

**DOI:** 10.1038/srep46277

**Published:** 2017-04-11

**Authors:** David Lagorce, Dominique Douguet, Maria A. Miteva, Bruno O. Villoutreix

**Affiliations:** 1 INSERM, U973, Université Paris Diderot, Sorbonne Paris Cité, Paris, France; 2 CNRS UMR7275, Institut de Pharmacologie Moléculaire et Cellulaire, Université Côte d’Azur, Valbonne, France

## Abstract

The modulation of PPIs by low molecular weight chemical compounds, particularly by orally bioavailable molecules, would be very valuable in numerous disease indications. However, it is known that PPI inhibitors (iPPIs) tend to have properties that are linked to poor Absorption, Distribution, Metabolism, Excretion and Toxicity (ADMET) and in some cases to poor clinical outcomes. Previously reported in silico analyses of iPPIs have essentially focused on physicochemical properties but several other ADMET parameters would be important to assess. In order to gain new insights into the ADMET properties of iPPIs, computations were carried out on eight datasets collected from several databases. These datasets involve compounds targeting enzymes, GPCRs, ion channels, nuclear receptors, allosteric modulators, oral marketed drugs, oral natural product-derived marketed drugs and iPPIs. Several trends are reported that should assist the design and optimization of future PPI inhibitors, either for drug discovery endeavors or for chemical biology projects.

Protein-protein interactions (PPIs) represent an essentially untapped source of potential targets for therapeutic interventions. The modulation of PPIs by low molecular weight chemical compounds, particularly by orally bioavailable molecules (i.e., the most convenient, safest and least expensive way to deliver drugs), would be very valuable in numerous disease indications^1^,^2^,^3^,^4^,^5^,^6^,^7^,^8^,^9^,^10^. However, it is known that direct orthosteric PPI inhibitors, as they most often bind to relatively flat surfaces displaying to 3–5 small subpockets, tend to have some physicochemical parameters that are correlated to poor PK/PD properties and in some cases to poor clinical outcomes^11^,^12^,^13^,^14^,^15^,^16^,^17^,^18^,^19^,^20^,^21^,^22^,^23^,^24^,^25^. Along the same line of reasoning, the discovery of small molecule chemical probes is critical to gain additional fundamental knowledge about the importance of PPI interactions in the health and disease states. For these probes to be of interest, they also have to fulfil some ADMET property requirements.

The analysis of thousands of PPI inhibitors (iPPIs) (hits or molecules that went through optimization cycles) reported in several databases^15^,^26^,^27^ indicated that these compounds have in general a high lipophilicity (analyzed via log P calculations) and a high molecular weight (MW), properties that are usually not favorable to the development of oral drugs (although there are numerous exceptions to these rules^11^,^28^,^29^). While the current state of the art investigations performed on iPPIs have essentially focused on physicochemical properties^5^,^14^,^15^,^26^,^27^,^30^,^31^,^32^,^33^, in the present study, we move beyond these classical physicochemical properties (PC) to also predict several Absorption, Distribution, Metabolism, Excretion and Toxicity (ADMET) parameters using online servers and established commercial packages^34^. In order to outline iPPIs features, computations were carried out on eight datasets collected from several databases^15^,^26^,^35^,^36^,^37^. These datasets involve compounds targeting enzymes, GPCRs, ion channels, nuclear receptors, allosteric modulators, oral marketed drugs (OMD), oral natural product-derived marketed drugs (NPD) and iPPIs. As drug-likeness property guidelines were historically derived from datasets that did not include natural product molecules^38^,^39^, we decided to extract NPD from the OMD group in an attempt to gain additional insights on this particular set of compounds. The present study highlights several trends and properties that could be considered to design future PPI inhibitors, either for drug discovery endeavors or for chemical biology projects.

## Results and Discussion

All datasets were prepared and curated using the same protocol including a filtering step that selects subsets of diverse and representative molecules (see the Methods section). The physicochemical (PC) and ADMET properties of the different datasets were then computed and compared.

### Structural and physicochemical properties

Several research groups have investigated the relationships between PC properties, potency and the ADMET profile of small compounds^23^,^40^,^41^,^42^,^43^,^44^. The reasoning behind is that PC properties can act on, for instance, efficacy, safety or metabolism. In addition, small molecule drug candidates and chemical probes must be sufficiently soluble and permeable for experimental assays and to reach their site of action and engage the primary targets. PC properties can also act on other molecular events as it has been shown that target families can be partially differentiated on the basis of PC (e.g. GPCR ligands’ mean MW and log P values were found to be 573Da and 4.8, respectively while for ligands of nuclear receptors the mean MW and log P were calculated to be 482Da and 6.9^45^). Thus, a better understanding of PC parameters may also assist the design of compounds that could bind multiple biological targets and display interesting polypharmacology profiles, a situation that should be beneficial to the treatment of diseases with complex etiologies^45^. The computed PC properties included in our study are: MW, hydrogen bond donors and acceptors (HBDs and HBAs), log P, log D, the topological polar surface area (TPSA), water solubility, the number of formal charges at pH 7 (charges) and rings, the maximum size of rings, the topology investigated by the analysis of rotatable bonds, a measure of molecular complexity and the number of stereocenters^20^,^23^,^40^,^41^,^42^,^43^.

MW is an important property in small molecule drug discovery^20^,^21^. Undoubtedly, this property can impact various molecular events such as absorption, bile elimination rate, blood brain barrier penetration, interactions with targets (on- and off-targets) while it is also commonly monitored during the compound optimization steps^22^,^23^,^24^,^25^. Our analysis reveals a global trend where iPPIs have on average the highest mean MW (521Da; 95^th^ percentile: 731Da) as compared to the other datasets (Table 1). Further, the 95% confidence intervals (CI) for the difference between the MW means of the different datasets was computed (Table S1) and we noted that the iPPIs mean is statistically significantly different from the means of the other subsets (p < 0.05). This confirms a well-known tendency of iPPIs that has been previously discussed^15^,^26^,^27^,^33^. The “iPPI MW challenge” might however be partially overcome in the coming years by developing dedicated compound collections that would be enriched in molecules with some specific 3D characteristics and distribution of properties in space important for binding at protein-protein interfaces but independent from properties such as MW or log P^46^,^47^.

Lipophilicity, characterized here by computed log P and log D values, plays a crucial role in determining several ADMET parameters as well as potency. For instance, solubility and metabolism are more likely to be compromised at high lipophilicity values whereas permeability could be decreased when this property is too low^48^. Very hydrophilic compounds are usually not able to diffuse passively through membranes, as they hardly enter the hydrophobic interior of the lipophilic bilayer while highly lipophilic compounds may poorly permeate membranes as they may get trapped into that bilayer. Furthermore, it has been reported that target promiscuity as well as toxicity issues like hERG inhibition, phospholipidosis or cytochrome P450 (CYP) inhibitions are more likely to be problematic for compounds with high lipophilicity values^22^,^48^. Our analysis shows that iPPIs and nuclear receptor compounds have a higher mean log P (4.8; 95^th^ percentile: 7.8 and 7.9, respectively) (Table 1 and S1) than the other datasets. Yet, while the two 95% CI overlap (iPPI and nuclear receptor compounds, Table S1), the difference between the mean log P values are statically significant. This trend follows for log D values (log P corrected for pKa of ionizable groups) with the highest values for the nuclear receptor compounds and iPPIs (mean log D 3.8 and 3.5, respectively, yet the difference between the two subset means is not statistically significant). Our data are similar to previously published ones indicating that our datasets are relevant in representing the different compound classes^20^,^21^,^49^.

HBAs and HBDs are other important parameters related to compounds polarity and permeability^50^. For instance, by analyzing PC fluctuation of marketed oral drugs over time^51^,^52^, it was found that MW and HBAs have significantly increased, whereas lipophilicity and HBDs showed relatively limited changes. These results suggest that HBDs count may be more crucial than HBAs count^20^,^53^ for drug development and may be related to efforts to enhance bioavailability and membrane permeability^51^. Indeed, it was shown that compounds containing more HBAs with less HBDs have favorable profile for both these parameters^22^,^53^. This is consistent with previously reported notes mentioning that HBDs are often the “enemy of medicinal chemists” (i.e., large number of HBDs could be the cause of poor permeability, absorption and bioavailability)^53^,^54^. Our analysis reveals that NPD molecules display the highest mean count of HBDs (2.8; 95^th^ percentile: 7) than the other populations while the mean HBD count for iPPIs was found to be 2.1 and significantly different from those of the other datasets (Table 1). Indeed, we note a mean value of 1.7 for the OMD dataset, highly similar to the value reported in ref. 21.

Regarding HBAs, we note that the enzyme dataset and iPPIs have higher values (both datasets are however not statically different) with a score of 7 (95^th^ percentile: 13 and 11, respectively).

The polar surface area (PSA) or the related topological surface area^55^ (TPSA) is another commonly investigated descriptor related to hydrogen bonding (oxygen and nitrogen atom count) that is important for permeability estimation and oral bioavailability^20^. Numerous predictive models show that these properties decrease when TPSA increases^56^,^57^ and especially in the case of CNS permeation by passive diffusion where TPSA must be below 80 Å^2^ 17,58,59. Our analysis shows that enzyme inhibitors tend to have the highest mean TPSA value (108 Å^2^; 95^th^ percentile: 202 Å^2^) while iPPIs are at 101 Å^2^ (statistically significantly different from the enzyme datasets (Table 1 and S1).

The ionization state of a molecule (acidic, basic…) plays either a beneficial or detrimental role on ADMET depending on the property involved^22^. Here, we investigated the formal charges of the compounds and observed that OMD, GPCRs and iPPIs tend to have more charged atoms (mean 0.7; 95^th^ percentile: 2) (Table 1). Note that iPPIs are statically similar (p-value > 0.05) from OMD, GPCR, ion channels and NPD.

TPSA can be used in combination with rotatable bond count to reflect molecular flexibility and it was proposed that bioavailability in rat decreases when the number of rotatable bonds and TPSA increase^56^. In our analysis, we find that iPPIs have the highest mean of rotatable bond count (7; 95^th^ percentile: 12; p < 0.05) (Table 1).

Molecular complexity is another property known to influence events such as solubility, oral bioavailability, permeability, promiscuity and clinical progression^60^,^61^,^62^. This measure accounts for the number of rings and aromatic rings, the fraction of carbons that are sp^3^ hybridized (Fsp^3^) or the number of stereocenters (these properties were computed by FAF-Drugs3^63^). For example, more than three aromatic rings in a molecule correlate with poorer compound developability and an increased risk of toxicity (hERG and CYP inhibition)^61^. Further, as aromaticity increases log P and affinity for albumin, it decreases the aqueous solubility as well as the free (non-bound) form species^62^. In our analysis, we note that iPPIs have the highest aromaticity (p < 0.05) with a mean count of aromatic rings of 3.3 (95^th^ percentile: 5) (Table 1). The average Fsp^3^ value has been shown to positively correlate with success in drug development as compounds with the highest Fsp^3^ values are likely to succeed at each stage of drug discovery^61^. Further, this topology descriptor may impact promiscuity and safety since it was found that promiscuity decreases as a Fsp^3^ increases^60^, although this observation was not confirmed in a recent study^64^. In their seminal work, Lovering *et al*. showed that the average Fsp^3^ was 0.36 for discovery compounds and increased to 0.47 for approved drugs. In our analysis, we confirm this observation as for OMD, the mean Fsp^3^ value is 0.4 (95^th^ percentile: 0.8) while it is 0.3 for iPPIs (statically different from the other populations p < 0.05) (Table 1). This observation suggests, as mentioned in^46^,^47^,^65^,^66^,^67^, that tri-dimensionality measured by computing Fsp^3^ values is a parameter that will need to be improved for the development of novel iPPIs.

**Figure 1:**
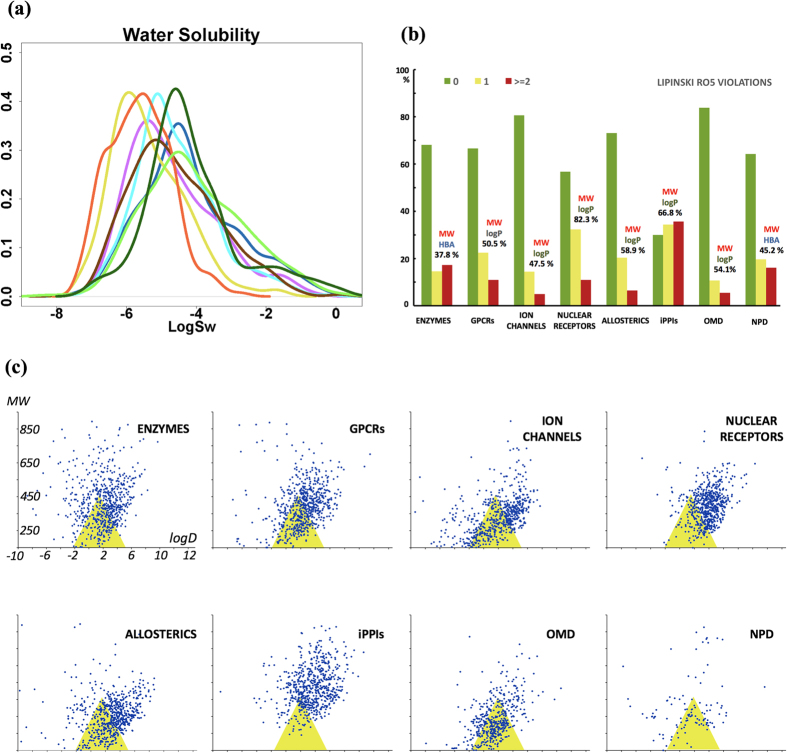
Physicochemical properties. **(a**) Solubility prediction: Kernel density estimation for water solubility (ordinate = density), expressed by the log S computed by the pkCSM server^72^. Enzymes (light-blue), ion channels (blue), GPCRs (purple), nuclear receptors (yellow), allosteric modulators (brown), iPPIs (orange), OMD (light green) and NP (dark green). **(b**) Rule of 5: Histogram distribution of the number of violations of the Lipinski’s RO5. For each dataset we provide the most frequent descriptor pair (and frequency) involved in the RO5 violation. **(c**) Golden Triangle: Golden Triangle representation for each dataset. Molecules (blue points) within the golden triangular area are more likely to be more permeable and have a low clearance. In the Golden Triangle study, it was reported that in the center of the triangle (log D 1.5, MW 350), 25% of the compounds would pass permeability and clearance criteria.

Solubility in intestinal fluid is another important property of oral drugs since insufficient solubility may limit the intestinal absorption through the portal vein system^22^,^68^,^69^. It is known that the development time of poorly soluble molecules tend to require two extra years and ultimately these compounds may lack efficacy due to a lack of exposure^20^. A strong relationship between solubility and lipophilicity has also been discussed previously^22^,^38^,^48^,^70^,^71^. The dataset with the highest mean solubility value, expressed as log S, is OMD (−4.07 and light green curve in Fig. 1) followed by NPD (−4.15) > ion channels (−4.2) > enzymes and allosteric modulators (−4.61) > GPCRs (−4.68) > nuclear receptors (−5.37) and iPPIs (orange line in Fig. 1) with the lowest mean log S value of −5.62. We also note that solubility on average decreases as MW and log P values increase (computed using the pkCSM server^72^). Solubility is thus another property that will have to be improved for the design of iPPI candidates and iPPI focused compound collections.

### Drug-likeness rules based on physicochemical properties

Several rules were developed in order to guide the selection of compounds in the early phases of drug discovery or to prepare chemical compound libraries suitable for drug discovery or chemical biology. Among the first applications of combined PC properties in drug discovery, the rule of 5 (RO5) was formulated in 1997 by Lipinski and colleagues^38^. The RO5 was derived from the analysis of orally available drugs and clinical candidates but excluded compound classes such as antibiotics, antifungals, vitamins and cardiac glycosides. The RO5 states that a compound is more likely to be membrane permeable and easily absorbed via passive diffusion in human intestine if it matches the following criteria log P ≤ 5; MW ≤ 500; HBAs (O + N atom count) ≤ 10 and HBDs (OH + NH count) ≤ 5. The RO5 suggests that molecules whose properties fell outside some boundaries, would be less likely to be orally absorbed. These defined cutoffs were chosen to capture ~90% of the ranges for the four calculated properties and in the original article, it was mentioned that the rule aimed at passive permeation estimation and is violated when two or more rules were broken. As a global trend, our analysis shows that 83% of OMD do not violate the RO5 > ion channels (81%) > allosteric modulators (73%) > enzymes (68%) > GPCRs (67%) NPD (64%), while only 30% of iPPIs inhibitors displays no violation. If we analyze the iPPIs subset which scores at least two violations (35%), the main pair of descriptors involved in RO5 violations is MW-log P (Fig. 1) with a prevalence of 67%. This observation is in line with results reported above as iPPIs are larger and more lipophilic than the other populations.

Another application of the combined analysis of PC properties is the so-called Golden Triangle, a visualization tool to help the simultaneous optimization of absorption and the clearance of drugs^73^. The approach was suggested to help select molecules that should be potent, metabolically stable and permeable drug candidates. When plotting MW versus log D (at pH 7.4) for a series of molecules, it was noticed that compounds with favorable permeability and low clearance were concentrated within a triangular shaped area, called the Golden Triangle. This study revealed that *in vitro* permeable and low clearance compounds are concentrated within a triangular area with a log D base-line ranging from −2.0 to 5.0 at MW = 200Da and a MW apex at 450Da. These properties were computed for our datasets (Fig. 1) and plots show that iPPIs hardly overlap the Golden Triangle with only 10.4% of the compounds fitting in the triangle. Similarly, a few 18.4% of the nuclear receptors compounds fit that triangle while the other populations have a better match (above 30% with the OMD subset scoring 50%).

### Absorption

Absorption can be conceived in simple terms as the process of movement of a drug from an extravascular site of administration into the systemic circulation. This process is indeed very complex and depends on numerous parameters^13^,^22^ of which permeability and compound solubility are crucial ones. As we already analyzed the solubility parameter in the previous section, we pursue here with permeability. Considering oral drugs, once they reach the gastro-intestinal tract, they must be able to move through biological membranes to enter the systemic circulation. Permeation can occur via transcellular diffusion, paracellular diffusion, and transporter-mediated mechanisms, with the former often being mimicked in the laboratory using artificial membrane assays, such as PAMPA variant, and the latter using MDCK or Caco-2 cell lines^22^. The permeability prediction was carried out on our datasets with StarDrop v6.1^74^ updated with a partial least square model built on Nordqvist *et al*. Caco-2 permeability data^75^ (see Supplementary information Fig. S1). In general, a compound is considered to have a high Caco-2 permeability if it has a Papp A → B > 8 · 10^−6^ cm/sec. Our results show that allosteric modulators, ion channels and nuclear receptors are those which possess the best-predicted permeability values (mean log Papp −4.9; ~ Papp A → B = 12.5 · 10^−6^ cm/sec) > GPCRs (−4.92) > OMD (−4.97) > NPD and iPPIs (−5.09) > enzymes inhibitors (−5.18; ~Papp A → B = 6.6 · 10^−6^ cm/sec). Interestingly, these predictions indicate that albeit iPPI compounds could be improved with regard to this property, they are not very different from the other datasets.

### Distribution

The distribution of drug refers to the distribution of the compound throughout different compartments within the body. Some parameters that can be investigated in silico with some degree of accuracy include blood–brain barrier (BBB) penetration or central nervous system (CNS) penetration and P-glycoprotein (P-gp) efflux. Moreover, because only the free (unbound) drug is available to interact with the protein target, its interaction with plasma proteins has to be monitored during the drug discovery process^18^.

### Plasma protein binding

**Figure 2:**
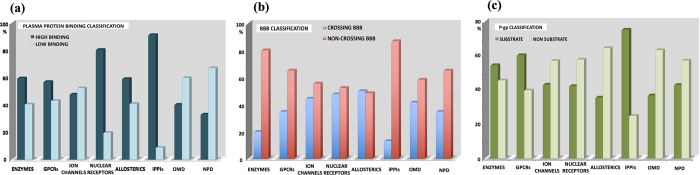
Some properties related to distribution. (**a**) Plasma protein binding: Plasma protein binding classification computed by StarDrop v6.1^74^. Compounds with less than 90% bound were classified as low binding molecules while the others are high binders. (**b**) Blood-brain-barrier penetration: Blood-brain-barrier penetration (BBB) classification predicted by StarDrop v6.1^74^. **(c**) P-gp prediction: P-gp inhibitors classification predicted by StarDrop v6.1^74^.

In Fig. 2, human plasma protein binding (PPB) values were categorized for each dataset, and predicted using a proprietary QSAR random forest model implemented in StarDrop v6.1^74^. It can be seen that the iPPIs subset displays high binding capacity (90%) and this observation holds for nuclear receptors (80%), while OMD scores around 45–50%. For example for iPPIs, this is consistent with high MW, log P^22^ and aromaticity^62^. This property may not be problematic for the development of iPPIs as most of the approved drugs have a high PPB value (>50%) and because an equilibrium exists between the free and unbound state (the complex dissociation being proportional to the disappearance of the free form).

### Central nervous system penetration

It is known that a high penetration is needed for most of the drugs that need to enter the central nervous system (CNS). A molecule must first cross the blood-brain barrier (BBB) with transcellular passive diffusion and/or active transport mechanisms^76^. However, BBB penetration should be minimized for non-CNS drugs to reduce the possibility of undesired pharmacological events and potential neurotoxicity. It has been suggested that, overall, compared to non-CNS drugs, CNS drugs tend to be more lipophilic (a log D value in the range 1–3 is recommended), more rigid, have a lower MW (≤450 Da), fewer hydrogen-bond acceptors (≤5), fewer formal charges (particularly negative charges), and a lower PSA (≤80 Å^2^)^59^. Here, the classification model of StarDrop v6.1^74^ categorized iPPIs as the dataset with the highest number of compounds (87%) which are predicted to not penetrate the CNS as compared to the other datasets following the order: enzymes (80%) <GPCRs and NPD (65%) <OMD (58%) <ion channels (55%) <nuclear receptors (52%) and allosteric modulators (48%) (Fig. 2). If we take into account the three cutoffs MW (≤450), TPSA (≤80) and HBAs (≤5), then only 11% of the iPPI dataset have CNS-like properties.

### P-glycoprotein efflux

Transporters play numerous roles in ADMET events, and P-gp is an important member that belongs to the ATP-Binding Cassette superfamily. These proteins use ATP as an energy source, allowing them to pump substrates against a concentration gradient^13^,^77^,^78^,^79^. P-gp is by far the most well-studied drug transporter and it is found in cells throughout the body, including those lining the intestine and the blood-brain barrier^22^. P-gp is believed to play an important role in defining the extent of distribution of drug molecules as a result of its ability to remove/extract a structurally diverse range of molecules from different compartments in the body. This transporter and some related others can reduce drug accumulation in certain tissues^22^,^79^. In addition, if a drug is subject to significant P-gp efflux, its distribution, absorption and elimination could be altered by P-gp inhibitors and evidence for drug-drug interactions due to inhibition of P-gp have been reported in several human clinical studies^80^. MW and log P are important PC parameters for P-gp efflux and, in general, when the MW increases, the P-gp efflux increases. P-gp efflux is reduced for molecules with a log P < 3 or > 5^22^. In order to evaluate this property, we decided to predict molecules that could act as P-gp substrates with the statistical model implemented in StarDrop v6.1. We note a higher proportion of P-gp substrates for the iPPIs dataset (75%) compared to GPCR (60%) > enzymes (54%) > ion channels and NPD (43%) > nuclear receptors (42%) > OMD and allosteric modulators (36%) (Fig. 2).

In conclusion for this section, our study shows that iPPIs are predicted to have high Plasma Protein Binding, to not penetrate the CNS and to be potential/likely substrates for P-gp transporters.

### BDDCS

**Figure 3:**
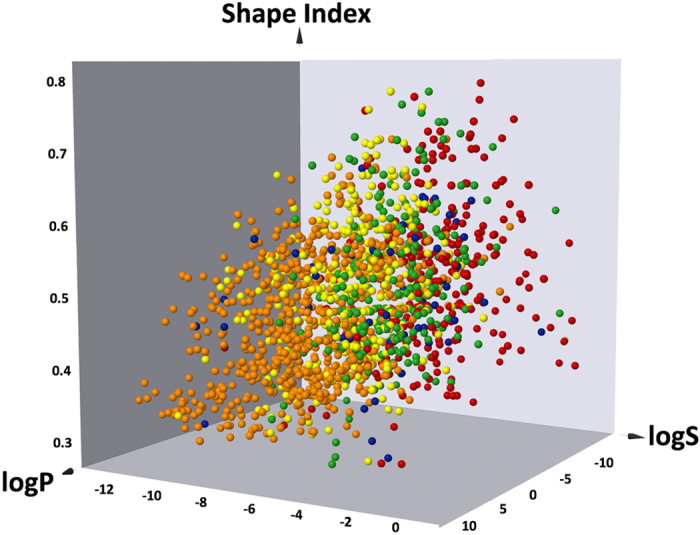
BDDCS. Mapping of the iPPI population (orange) in a trivariate scatterplot (generated with DataWarrior v4.4.3^85^ with log P versus water solubility (log S) versus compound shape index. The shape index is computed by calculating the shortest distance between any two non-hydrogen atoms of the molecule. The distance between any two atoms is the number of atoms in the chain including both chain ends. The longest of these shortest connections divided by the number of non-hydrogen atoms of the molecule gives the shape index. The Biopharmaceutical Drug Disposition Classification System classes are displayed for comparisons: class I in green, class II in yellow, class III in red and class IV in blue.

The Biopharmaceutics Drug Distribution and Classification System (BDDCS) attempts to split compounds into four classes based on their permeability and solubility properties. This system can be helpful in predicting the effects that drug transporters will have on a drug’s pharmacokinetic profile and this classification may assist some steps of the drug discovery process^81^. The BDDCS is a modification of the Biopharmaceutics Classification System (BCS) proposed by Amidon *et al*.^82^ that is based on the experimentally determined permeability and solubility characteristics of a drug compound. In the BDDCS system, Class I = High solubility − High permeability − High extent of Metabolism; Class II = Low solubility − High permeability − High extent of Metabolism; Class III = High solubility − Low permeability − Poor Metabolism; and Class IV = Low solubility − Low permeability − Poor Metabolism. There are many additional applications of the BDDCS system such as trying to predict drug-drug interactions, elimination routes, central nervous system exposure, toxicity, and environmental impacts of drugs to cite a few of them^83^. Here we decided to compare our datasets with over 1000 drugs with known BDDCS classes as compiled by Benet *et al*. and Hosey *et al*.^83^,^84^. To this end, we used the DataWarrior v4.4.3 package^85^ to map compounds on a trivariate plot comprising log P, water solubility (log S) and the compound shape index (Fig. 3; the shape index is further described in the figure legend). First, the generated plot shows that the combination of these three descriptors can cluster the four BDDCS classes. Although it is a visual representation of the data and not a prediction model, we note that the iPPIs dataset (orange dots) overlaps primarily class 2 molecules (yellow dots) suggesting that many iPPI compounds belong to the Low solubility – High permeability – High extent of Metabolism class 2. We also analyzed the other datasets but no clear tendencies were noticed except that the nuclear receptor group better overlaps the properties of BDDCS class 2 compounds while OMD are distributed over the four classes (data not shown).

### Metabolism, metabolic stability and clearance

Metabolism is the biotransformation of drugs and xenobiotic compounds to facilitate their excretion. Metabolic liability can lead to a number of issues, such as poor bioavailability due to enhanced/high clearance; toxic effects caused by reactive metabolites and drug-drug interactions (DDIs) including enzyme inhibition, induction, and mechanism-based inactivation^86^. Metabolic processes are mainly catalyzed by the so-called phase I (oxidation, reduction, and hydrolysis) and II (sulfo-conjugation among others) enzymes, which are, for the most part, produced in the liver. In order to investigate these events, several experimental approaches can be used. For example, assaying hepatic microsomes that contain hepatic enzymes to test the metabolic stability of a molecule. Other assays with hepatocytes or recombinant enzymes usually provide complementary information^87^. Alternatively, several in silico methods have also been published^18^,^22^,^23^,^88^,^89^,^90^,^91^. Finally, the drug elimination process called clearance generally results from both liver metabolism and excretion, mostly performed by the kidneys. The clearance can be estimated using *in vivo* animal models but also by *in vitro* measurements on liver microsomes or hepatocytes when investigating hepatic clearance alone.

### Metabolic stability and total clearance

Metabolic stability can be defined as the susceptibility of a chemical compound to biotransformation, and is expressed as *in vitro* half-life (t1/2) and intrinsic clearance. The half-life for our datasets was predicted using StarDrop v6.1 updated with the human liver microsome stability model (mainly phase I enzymes) previously developed by Zakharov *et al*.^92^. No clear differences were noticed among the different datasets (see Supplementary information Fig. S2) suggesting that the presently available model is possibly not sufficiently accurate to evaluate this property. The total body clearance (the sum of different clearance mechanisms), just like metabolic stability, is known to be a complicated endpoint to model because it involves multiple enzymatic reactions and depends on factors such as the extent of plasma protein binding, the volume of distribution and the involvement of active transports across membranes^92^. The computation of total clearance as Log(CL_tot_) was performed with the pkCSM server^72^ which predicts the combination of hepatic clearance (metabolism in the liver and biliary clearance) and renal clearance (excretion via the kidneys). Some differences can be noticed between NPD, nuclear receptors and iPPIs (mean 0.57, 0.63 and 0.64 ml/min/kg respectively) and the other datasets, especially ion channels (mean 0.80 ml/min/kg) (Fig. S4).

### Toxicity

Attrition due to toxicity and clinical safety concerns is a major problem in drug discovery^93^,^94^,^95^. Toxicity is the degree to which a substance can damage an organism or substructures of the organism, such as cells and organs, and remains one of the most significant reasons for late-stage drug development failure. Early identification of toxicity would thus be very valuable^86^. Among the different kinds of toxicities, one can cite hepatic, hematologic and cardiovascular toxicity, but many other outcomes exist, for instance carcinogenicity, teratogenicity, reproductive toxicity, cytotoxicity, and phospholipidosis^96^,^97^,^98^. Toxicity mechanisms can be classified into various categories: pharmacophore-induced toxicity (e.g., human ether-a-go-go-related gene binding), structure-related toxicity (structural features and physicochemical properties allowing interactions at sites distinct from the intended target), metabolism-induced toxicity (e.g., electrophiles can react with nucleophilic functions in endogenous biomolecules and cause organ toxicity) and toxicity linked to dosage (monitored by experimental methods like the “Maximum Tolerated Dose”(MTD), “No Observable Adverse Effect Level” (NOAEL) or Oral Rat Lethal Dose (LD_50_)^99^. Furthermore, toxicity can also be caused by drug–drug interaction (DDI) which can lead to the withdrawal of drugs from the market^13^,^99^. Several types of DDI can occur and various in silico drug-drug interaction prediction engines have been developed^88^,^100^,^101^. For instance, in silico DDI assessments can be performed by estimating the possible binding of a compound to important proteins that participate in DDI such as CYP enzymes^102^ and transporters (e.g, P-gp)^93^,^103^. Overall, toxicity is investigated using various experimental approaches but in silico models can also help, although they are difficult to develop and tend to be more reliable when they focus on specific endpoints^93^.

### CYP P450s inhibitions

**Figure 4:**
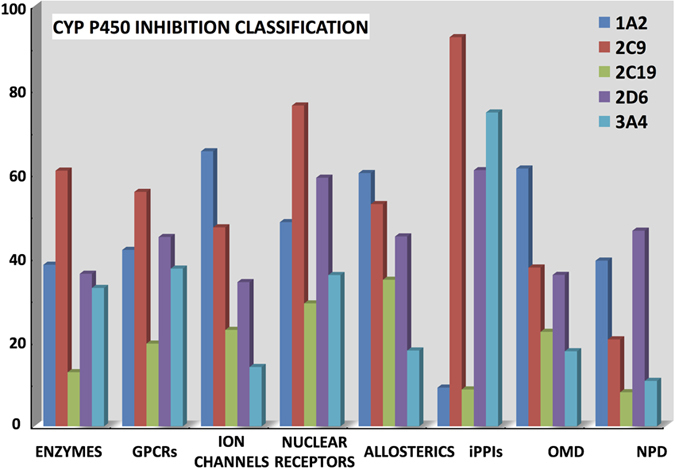
CYP P450s inhibition. Classification of inhibition of 1A2, 2C9, 2C19, 2D6 and 3A4 isoforms predicted by the pkCSM web-server^72^ and StarDrop v6.1^74^.

As the cytochrome P450 mono-oxygenase (CYP) enzymes superfamily plays a pivotal role in drug metabolism, they have been extensively investigated, especially 2D6, 2C9 and 3A4 which are the most important forms in human^13^,^91^,^104^. In order to estimate which compounds in our datasets may be binders for the CYP450s, we used StarDrop v6.1^74^ for the 2D6 and 2C9 isoforms while 1A2, 2C19 and 3A4 were investigated by using the binary classification of the pkCSM server^72^. A continuous random forest model is implemented in StarDrop to predict 2C9 pKi values while a classification model is present in the package for 2D6 (low (pKi < 5), medium (5 < pKi < 6), high (6 < pKi < 7) and very high (pKi > 7)). In the order to facilitate the analysis, we categorized the predicted 2C9 following the approach used for 2D6. These estimations suggest that iPPIs and nuclear receptor compounds tend to inhibit both these isoforms. Indeed, these populations score 60% for 2D6 and respectively 92% and 76% of iPPIs and nuclear receptors bind 2C9 (Fig. 4). We also noted that iPPIs are predicted to be strong inhibitors of the 3A4 isoform (75%) (Fig. 4). This subset of compounds displays a low level of inhibition for 1A2 and 2C19 (both 9%) while the other datasets are expected to inhibit mainly the 1A2 isoform (e.g., 65% for ion channels and 61% for OMD). These results have to be considered with cautions as pertinent prediction models are difficult to develop due to the complex molecular mechanisms involved in CYP inhibition^18^, but it allows an overall comparisons among the different classes of molecules.

### Hepatotoxicity

**Figure 5:**
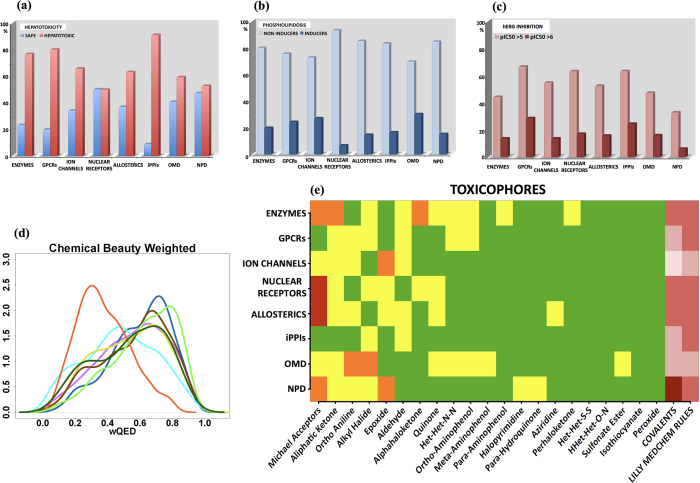
Some properties related to toxicity and structural alerts. **(a**) Hepatotoxicity classification: The prediction was performed with the pkCSM server^72^. **(b**) Phospholipidosis prediction: Classification of phospholipidosis inducers according to Przybylak *et al*. model^97^,^111^ (computed with FAF-Drugs3^63^). (**c**) hERG prediction: Classification for compounds that need a specific attention (pIC50 ≥ 5) and compounds which may exhibit some hERG toxicity endpoints (pIC50 ≥ 6). **(d**) QED estimation: Kernel density estimation for chemical beauty based on the weighted quantitative estimate of drug-likeness (wQED) computed by StarDrop v6.1^74^. Enzymes (light-blue), ion channels (blue), GPCRs (purple), nuclear receptors (yellow), allosteric modulators (brown), iPPIs (orange), OMD (light green) and NPD (dark green). **(e**) Toxicophores: Matrix plot of toxicophores detection computed by the FAF-Drugs3 web-server^63^. Frequency (%) colored as following: green < 0.4, 0.4 < yellow < 2, 2 < orange < 4, 4 < red < 6.2, 6.2 < light pink < 10, 10 < pink < 15, 15 < dark pink < 30, 30 < purple < 50.

Hepatotoxicity remains a major reason for drug withdrawal from pharmaceutical development and clinical use. Often, *in vivo* screening for hepatotoxicity is performed during the preclinical phases of the development process, however, more than 40% of compounds showing liver effects in humans did not present effects in previous animal studies^105^. In parallel, *in vitro* testing are also available through assays processed on primary human hepatocytes cultures, cultured immortalized cell lines like HepG2 or liver slices. As well, perfused livers testing can also be used in order to evaluate cytotoxic induced effects such as mitochondrial damage, oxidative stress, covalent binding and intracellular interaction with glutathione^106^. Some in silico predictive approaches have emerged with the MCASE program^107^ or QSAR models^108^,^109^. We here used the pkCSM server^72^ to investigate this toxicity endpoint. We note that iPPIs are predicted to be highly hepatotoxic (91%) unlike the nuclear receptor (50%) or OMD (40%) compounds (Fig. 5). However, enzymes and GPCRs also hit 80%. In comparison, 47% of the drugs listed in the LiverTox Database did not have evidence of hepatotoxicity including antineoplastic agents^110^.

### Phospholipidosis

Phospholipidosis (PLD) is an adverse drug reaction in response to cationic amphiphilic drugs (e.g., anti-depressants, antibiotics, and cholesterol-lowering agents) that leads to a lipid storage disorder due to the accumulation of polar phospholipids in the lysosomes (lysosomotropism)^97^. This accumulation of drug-phospholipid complexes within the internal lysosomal membranes induces an abnormal accumulation of multi-lamellar bodies (myeloid bodies) in tissues. This adverse side effect can for example affect the registration of new drug entities^111^,^112^. We computed this property using our online server FAF-Drugs3^63^ which applies the SMARTS-based model developed by Przybylak *et al*.^97^,^111^. It can be seen that the percentage of molecules that are predicted to be inducers follows the global trend: OMD (30%) > ion channels (27%) > GPCRs (24%) > enzymes (20%) > iPPIs (16.5%) NPD and allosteric modulators (15%) and then nuclear receptors with 7% of inducers (Fig. 5). A recent *in vitro* study predicted 24% of inducers in a set of small drug-like compounds including a high proportion of marketed drugs^113^ and is thus in line with our observation for this category of OMD molecules.

### hERG

Several types of cardiovascular toxicity issues have to be considered, but admittedly, promiscuous block of cardiac human ether-a-go-go-related gene (hERG) channels by a variety of structurally different low molecular weight drugs represents a major therapeutic challenge with profound impacts on human health^114^,^115^,^116^,^117^,^118^. The model implemented in StarDrop v6.1^74^ predicts that GPCRs, nuclear receptors and iPPIs have the highest levels of hERG pIC_50_ inhibition with a mean pIC_50_ of 5.3, while the other datasets are ranked as follow: allosteric modulators (5.1) > ion channels (5.05) > OMD (5) > enzymes (4.8) and NPD (4.45) (Fig. S3). With this model, if the value is above > 5, it is advised to experimentally test the binding as the compounds are likely to exhibit some toxicity endpoints related to this potassium channel^114^,^119^. We thus report a categorization histogram (Fig. 5) where one can see that 33% of NPD has pIC_50_ > 5 and 67% and 64% for GPCRs and iPPIs, respectively (both these subsets have 29% and 25% of compounds with pIC_50_ > 6).

### Oral Rat pLD_50_

The median lethal dose (pLD_50_) is a standard measurement of acute toxicity (dose causing 50% death of the treated animals when administered during a given period) used to assess the relative toxicity of different molecules. Acute toxicity describes the adverse effects of a substance that occur within a short period after exposure and is an important indicator of the drug safety assessment typically performed during the first stages of toxicological investigations of unknown substances^86^,^120^,^121^. By computing this property with the pkCSM server^72^, we note a small increase of the mean pLD_50_ for iPPIs with a –log(LD_50_) equal to 2.9 compare to the others datasets (see Supplementary information Fig. S5). A mean LD_50_ of 2.7 is computed for ion channels while for OMD and NPD the values are lower, 2.5 and 2.4 respectively.

### Structural alerts and PAINS

Structural alerts (SA) or toxicophores (between 30–200 described chemical moieties) can, directly or upon bioactivation, be linked to toxicity^94^,^95^,^96^,^122^,^123^,^124^. In addition, several chemical groups and compounds have been described to interfere with biological assays, the so-called PAINS compounds^125^,^126^,^127^,^128^,^129^,^130^,^131^ (pan-assay interference compounds). These are compounds that have been observed to show activity in multiple types of assays, often by interfering with the assay readout rather than through specific compound/target interactions (e.g., covalent binding, metal chelation, redox reactivity, aggregation, fluorescence interference…)^127^. Some structural motifs can form covalent protein/DNA modification and subsequent downstream adverse outcomes (i.e. CYP inhibition, *in vitro* genotoxicity, carcinogenicity or *in vivo* hepatotoxicity). The covalent modification of endogenous biomolecules, which is the primary issue, could be linked to the inherent chemical reactivity of a SA and/or alternatively, appears upon bioactivation through the generation of a reactive metabolite (e.g. anilines)^94^,^95^,^123^. Here we searched for SA and PAINS with our online server FAF-Drugs3^63^, and we show that iPPIs do not specifically contain many SA (less than 2% and only 2 major chemical moieties are found: alkyl halide and aldehyde (Fig. 5). The datasets that contain more SA are enzymes (15%), OMD (12%), allosteric modulators (11%) and nuclear receptors (9%). In Fig. 5, results are ordered gradually from SA motifs that are the most retrieved to the ones that are less found in our datasets starting by the Michael acceptors (18%) then quinone categories (3%), ortho-anilines (7%) and epoxides (6%). Michael acceptors are electrophilic agents which may form covalent bonds with nucleophilic sites on proteins and DNA molecules that can lead to carcinogenicity. Figure 5 also reveals that NPD are compounds that embed the highest number of substructures (47%) potentially involved in covalent binding like the β-lactam ring while nuclear receptors rated 25% and enzymes 23%. Further AMES mutagenicity predictions were also performed and some variations are noticed for the different datasets (Fig. S6).

Regarding molecules that may interfere with assays, the major reasons can be compounds that are not specific to the target (e.g. promiscuous compounds and aggregators^132^, frequent hitters^133^, some PAINS^127^) and/or (2) compounds that perturb the assay or detection method (e.g. colored or fluorescent molecules and aggregators). In both cases, such molecules are usually poor starting points for lead optimization programs and can cause an expenditure of money and loss of time without major benefits^126^,^127^,^129^,^130^,^134^,^135^. However, PAINS substructures search like PC guidelines must be applied carefully when selecting candidates, because, there are many observed exceptions to these rules. Blindly applying such rules can discard from development some interesting molecules. For PAINS and SA, it may be better to keep potential metabolic liabilities (easy to substitute during the optimization phases) rather than to discard a valuable diversity subspace^136^,^137^. We searched for PAINS compounds with the FAF-Drugs3 sever^63^ that embeds the original PAINS definitions^127^. The results indicate that iPPIs are the dataset which score highest level (22%) for the presence of PAINS (Fig. S7). Catechol_A (12%) and quinone_A (8.5%) (see Supporting information in ref. 127) are the most frequent PAINS found in our datasets. While quinone_A substructure is not found in the iPPI dataset, catechol_A is part of the highest retrieved substructure in this population (5%), together with anil_NH_alk_B (5.6%) and sulfonamide_A (3.3%).

### Toxicity based on rules combining PC properties and/or structural alerts

Whereas several guidelines combining physicochemical parameters have been reported to predict ADMET properties, some rules have also been suggested to apply directly to toxicity. For instance, the importance of combining two PC properties has been recently reported^138^. In that study, the authors investigated the toxicological outcomes of 245 compounds in development at Pfizer and found that compounds with log P > 3 and TPSA < 75 Å^α^ were six times more likely to show an adverse event in a rat or dog *in vivo* safety study than a compound with log P < 3 and TPSA > 75 Å^2^. It was also suggested that the combination of high log P with a low TPSA increases the likelihood of promiscuous binding to off-targets. While this rule could be used as guideline and help to select molecules for optimization, it has to be used with caution as recent analyses do not confirm the initial report^64^,^139^. This again suggests that strictly adhering to rules could result in missed opportunities^93^. A potential reason for these discordances could be due to the differences in the dataset composition (preclinical, phase I and/or drug candidates), the software programs used to calculate properties, or the reported information that described the origin of toxicity (related to the primary target or to the compound itself)^64^. Our analysis shows that more than 50% of the nuclear receptors compounds and 45% of allosteric modulators fit in the problematic region of the Pfizer 3/75 rule while 30% and 26% of OMD and iPPIs populates this region, respectively (data not shown).

Recently, Bickerton *et al*. reported a quantitative measure of drug-likeness^39^ based on a concept of desirability called the quantitative estimate of drug-likeness (QED). The novelty of this approach stands on the fact that this estimation does not only rely on PC parameters but it also involves searching for structural alerts (see below). QED ranks compounds according to their similarity to marketed drugs by a continuous measure of drug-likeness estimated by calculating eight important properties: MW, log P, number of HBDs and HBAs, TPSA, number of rotatable bonds, number of aromatic rings and number of structural alerts. Thus, this approach does not strictly use a yes–no cut-off filter above which compounds are disqualified, and probably minimizes probable estimation errors in individual computational predictors. A recent analysis places drugs’ QED median value at 0.65^103^ while a value of 0.67 was proposed for the so-called attractive or promising compounds^39^. The calculations performed with StarDrop v6.1^74^ indicates a median QED value of 0.65 for the OMD subset (Fig. 5). Not surprisingly, iPPI compounds (orange line) showed a clear difference with the other populations because several PC descriptors used to estimate QED are obviously shifted in the wrong direction. We note that ion channels display similar values than OMD (0.63) and that enzyme binders have lower scores of about 0.48. Overall, iPPIs scored a QED comparable to that obtain for the unattractive compounds reported by Bickerton *et al*. (0.35) indicating that chemistry efforts will be required to improve the quality of the next generation of iPPIs.

Likewise, a recently reported in silico approach that helps the selection of compounds that could enter open drug discovery programs is the Eli Lilly MedChem Rules package^140^. These rules involve queries for about 275 structural alerts including compounds/substructures that are unstable, reactive, interferent, promiscuous, and compounds with risks of toxicity or poor *in vivo* stability. According to these rules, we note that, whatever the subsets, between 15 to 30% of the molecules do not comply (regular mode), except for OMD and ion channels compounds where only 10 to 15% would be discarded (Fig. 5).

Overall, these toxicity predictions suggest that some iPPIs are associated with toxicity alerts. Indeed, (1) PPI inhibitors have the highest predicted levels of hERG pIC_50_ inhibition and inhibit several CYP enzymes, (2) are predicted to be hepatotoxic and (3) may cause few acute toxicities in rats. On the other hand, these compounds (1) do not embed many SA, (2) are less inducers of phospholipidosis than OMD and (3) do not induce mutagenicity (Fig. S7). Regarding PAINS, four structural alert families have been found in iPPIs, suggesting that, in the future, compound collections dedicated to the design of such molecules should be flagged with PAINS filters.

## Conclusion

Inhibition of protein-protein interactions with small molecules using screening or repositioning strategies is of high interest for both, the development of new therapy and to explore novel molecular mechanisms involved in the health and disease states^1^,^2^,^3^,^4^,^5^,^6^,^7^,^8^,^9^,^10^,^141^. However, the design of iPPIs is challenging and we were here interested in the analysis of predicted PC and ADMET properties for these small molecules that we compared to other datasets containing molecules acting on other targets or molecular mechanisms. We first note that iPPIs possess borderline PC values in all calculated properties except for TPSA, HBDs and the number of stereocenters. iPPIs tend to violate the RO5 and are most often outside the Golden Triangle. With regard to absorption, our results predict a relatively good absorption for iPPIs as compared to the other datasets. Further, iPPIs membrane permeability is comparable to that of NPD compounds and better than that of enzymes. Regarding distribution, iPPIs are predicted to bind to PPB as nuclear receptors but this may not be a major issue as most OMD also binds significantly to PPB. Yet, for the time being, given the computed properties, it would seem difficult to develop small molecule protein-protein interaction inhibitors for CNS targets while there are obviously major needs in this area. iPPIs are potential P-gp binders and this property must be carefully monitored. Data visualization methods suggest that iPPIs could belong mainly to the class 2 molecules of the BDDCS system (low solubility, high permeability, high metabolism). Regarding metabolism, we noted no major differences for the calculated half-life and total clearance of iPPIs versus the other datasets. 2C9, 2D6 and 3A4 inhibition have been correlated to MW and log P with a contribution of the ionization state. Thus, considering values in Table 1, it is consistent that iPPIs and NR share these trends and could be highly metabolized or inhibitors of this family of enzymes. For the investigated toxicity endpoints, we note that the hepatotoxicity profile of iPPIs is high and this observation holds for cardiotoxicity investigated via hERG inhibition. iPPIs do not show significant phospholipidosis or mutagenic warnings while the mean LD_50_ is slightly superior to the other datasets. Regarding unwanted structural motifs (Structural Alerts and PAINS), known iPPIs do not contain many such substructures as compared to the other datasets. iPPIs are not well positioned in term of QED scores but are acceptable when considering the 3/75 toxicity prediction rule. Taken together, the reported data should help designing the next generation of iPPIs.

## Methods

### Datasets preparation

The iPPIs dataset (compounds with bioactivity below 30 μM) was built by merging all compounds from IPPI-DB^26^ minus bromodomain’s inhibitors while adding 89 compounds extracted from the TIMBAL database^15^ targeting menin-mixed lineage leukemia (MLL) and neuropilin, and 24 small molecule disruptors of the glucokinase–glucokinase regulatory protein interactions^142^. Regarding the “non-iPPIs” modulators, all datasets were created using the version 14 of the ChEMBL database categorization^36^ which is available at ftp.ebi.ac.uk/pub/databases/chembl/Allosterism. We extracted these molecules from the version 20 of the ChEMBL database^35^ with the highest ChEMBL confidence score of 9 and a bioactivity below 30 μM^143^. We formed one category of allosteric molecules (allosteric modulators of kinases, proteases, phosphodiesterases, phosphatases, nuclear receptors, ion channels and GPCRs) and four categories of orthosteric molecules (nuclear receptors, ion channels, GPCRs and enzymes (proteases, kinases, phosphodiesterases and phosphatases)). From the same database, we retrieved the non-allosterics oral bioavailable approved drugs (OMD) and we extracted from it the natural product-derived compounds (NPD). We removed from both these subsets putative allosterics modulators showing occurrences in the Allosterics ASD database^144^. Then, all datasets were treated following the same filtering and diversity search protocols. We performed with the FAF-Drugs3 web-server^63^ the selection of compounds within the 150 to 900 Da area (filtered subset). On these molecules, we applied a clustering protocol with the Accelrys Pipeline Pilot FCFP4 fingerprints (maximum Tanimoto coefficient of 0.2) where the centroid of each cluster was taken to build the diversity subset. In order to have a relatively similar number of chemically diverse compounds in each dataset, we kept the entire diversity subset when its amount was below 650 compounds, otherwise we proceeded a random picking after diversity searching (random subset). To insure that diversity or random subsets represent properly the original filtered subsets, we visualized the chemical space of each subset using the path-based fingerprints projection visualization tool of StarDrop 6.1^74^. In the same space we visualized the filtered subsets (light blue), the diversity subsets (red) and the random subsets (white) (see Supplementary information Fig. S8).

### PC computations

We used the FAF-Drugs3 web-server^63^ to compute physicochemical descriptors: number of rotatable bonds, rigid bonds, HBAs, HBDs, rings, charges (formal charges at pH 7), heavy atoms, carbon atoms, heteroatoms and stereocenters, MW, log P, log D (at pH 7), TPSA, maximum size of ring, number of rings and aromatic rings, flexibility, total charge and Fsp^3^ 61. We also derived the Lipinski’s RO5^38^, the Pfizer’s 3/75 rule^138^ and the Golden Triangle^73^. The estimation of the chemical beauty^39^ was carried out with StarDrop v6.1^74^ while the water solubility was computed with the pkCSM server^72^.

### ADMET predictions

Regarding the ADMET predictions, total clearance, CYP P450 inhibition and hepatotoxicity were computed with the pkCSM^72^ server. PPB, BBB crossing, CYP P450, P-gp classification, hERG inhibition and Rat LD_50_ estimation were obtained with StarDrop v6.1^74^. In addition, this software was updated with three free available add-ons: (i) a partial least square (PLS) model of the Caco-2 permeability developed by Optibrium Ltd. developers with experimental values^75^, (ii) a radial basis function (RBF SD) estimating the microsomal metabolic stability^92^ and (iii) a Optibrium Ltd. AMES mutagenicity model built with the StarDrop Auto-Modeller module^74^. Finally, the FAF-Drugs3 server^63^ predicted phospholipidosis by using the Przybylak *et al*. method^97^ and was used to detect PAINS and 154 structural alerts.

## Additional Information

**How to cite this article:** Lagorce, D. *et al*. Computational analysis of calculated physicochemical and ADMET properties of protein-protein interaction inhibitors. *Sci. Rep.* **7**, 46277; doi: 10.1038/srep46277 (2017).

**Publisher's note:** Springer Nature remains neutral with regard to jurisdictional claims in published maps and institutional affiliations.

## Supplementary Material

Supplementary Data

## Figures and Tables

**Table 1:** Median (Mdn), mean (M), standard error of the mean (SEM) and value at 95% percentile (P) for PC computations of all datasets.

	Enzymes				GPCRs				Ion Channels				Nuclear Receptors				Allosterics				iPPIs				OMD				NPD			
	Mdn	M	SEM	P	Mdn	M	SEM	P	Mdn	M	SEM	P	Mdn	M	SEM	P	Mdn	M	SEM	P	Mdn	M	SEM	P	Mdn	M	SEM	P	Mdn	M	SEM	P
MW	396	415	14	673	391	398	13	614	330	337	11	537	396	396	10	569	352	356	11	531	508	521	12	731	316	337	11	540	354	399	16	796
log P	3.1	3.1	0.2	6.4	3.7	3.5	0.2	6.4	3.1	2.9	0.2	5.9	4.6	4.8	0.2	7.9	3.6	3.6	0.2	6.6	4.8	4.8	0.2	7.8	2.8	2.9	0.2	5.7	2.1	2	0.3	6.2
log D (pH 7)	2.3	1.9	0.3	5.5	2.4	2.3	0.3	5.8	2.4	1.8	0.3	5.5	3.9	3.8	0.2	6.2	2.9	2.6	0.3	6.5	3.6	3.5	0.2	7.1	1.5	1.5	0.2	5.2	1.6	1.2	0.4	5.8
TPSA	99	108	5.2	202	69	78	4.6	148	64	71	4.1	149	68	71	3.1	115	64	71	4.1	130	95	101	4	180	70	72	3.8	136	90	103	6.5	214
Rotatable Bonds	5	6.5	0.5	16	6	6	0.4	12	4	4	0.3	10	5	5.5	0.4	12	5	5.6	0.5	14	7	7	0.3	12	5	5.3	0.4	11	3	3.9	0.3	10
HBDs	2	2.5	0.2	6	1	1.7	0.2	5	1	1.5	0.1	4	1	1.5	0.1	3	1	1.4	0.2	4	2	2.1	0.2	5	2	1.7	0.1	4	2	2.8	0.3	7
HBAs	6	7	0.3	13	5	5.6	0.3	10	5	4.9	0.2	9	4	4.5	0.2	8	4	4.8	0.3	8	7	7	0.2	11	5	4.9	0.2	9	5	6.5	0.4	15
HBDs + HBAs	9	9.5	0.5	19	7	7.3	0.4	13	6	6.5	0.3	12	6	6	0.3	10	5	6.1	0.4	12	9	9.1	0.3	15	6	6.6	0.3	12	8	9.3	0.6	19
Rings	3	2.7	0.1	4	3	2.7	0.1	4.5	2	2.1	0.1	3	2	2.4	0.1	4	2	2.2	0.1	4	4	3.7	0.1	5	2	2	0.1	4	1	1.6	0.1	3.5
Aromatic Rings	3	2.6	0.1	4	2	2.4	0.1	4	2	2.2	0.1	4	3	2.4	0.1	4	2	2.2	0.1	4	3	3.3	0.1	5	2	1.8	0.1	4	1	0.8	0.1	2.5
Stereocenters	0	1.2	0.2	5	1	1.4	0.2	5	1	1.3	0.2	4	1	1.5	0.2	7	0	0.8	0.1	4	1	1.6	0.2	5	1	1	0.1	4	5	5.7	0.4	18
Fsp^3^	0.3	0.3	0	0.7	0.4	0.4	0	0.7	0.3	0.3	0	0.8	0.3	0.3	0	0.8	0.3	0.3	0	0.7	0.3	0.3	0	0.5	0.4	0.4	0	0.8	0.6	0.6	0	1
Formal charges (pH 7)	0	0.5	0.1	2	1	0.7	0.1	2	0	0.6	0.1	2	0	0.4	0.1	1	0	0.5	0.1	2	1	0.7	0.1	2	1	0.7	0.1	2	0	0.6	0.1	2
