# Supplementary material for: Computational analysis of calculated physicochemical and ADMET properties of protein-protein interaction inhibitors
Source: Sci Rep. 2017 Apr 11;7:46277. doi: 10.1038/srep46277 (PMC5387685; doi:10.1038/srep46277)
Supplement: Supplementary Data [file srep46277-s1.pdf]

Supplementary Information

Computational analysis of calculated physicochemical and ADMET properties of protein-protein interaction inhibitors

David Lagorce[1], Dominique Douguet[2], Maria A. Miteva[1], Bruno O. Villoutreix[1]

[1]INSERM, U973, Université Paris Diderot, Sorbonne Paris Cité, Paris, France

[2]CNRS UMR7275, Institut de Pharmacologie Moléculaire et Cellulaire, Université Côte d'Azur, Valbonne, France

Correspondence to: Bruno Villoutreix

e-mail: Bruno.Villoutreix@inserm.fr

Keywords: ADMET, toxicity, chemical space, protein-protein interaction inhibitor, drug design, virtual screening

	Enzymes				GPCRs				Ion Channels				Nuclear Receptors				Allosterics				iPPIs				OMD				NPD			
	M	SEM	LB	UB	M	SEM	LB	UB	M	SEM	LB	UB	M	SEM	LB	UB	M	SEM	LB	UB	M	SEM	LB	UB	M	SEM	LB	UB	M	SEM	LB	UB
MW	415	14	405	427	398	13	388	407	337	11	328	345	396	10	388	403	356	11	348	365	521	12	513	530	337	11	326	347	399	16	369	428
logP	3.1	0.2	3	3.3	3.5	0.2	3.4	3.7	2.9	0.2	2.7	3	4.8	0.2	4.7	4.9	3.6	0.2	3.5	3.8	4.8	0.2	4.7	5	2.9	0.2	2.7	3	2	0.3	1.4	2.6
logD	1.9	0.3	1.7	2.1	2.3	0.3	2.1	2.5	1.8	0.3	1.6	2	3.8	0.2	3.6	3.9	2.6	0.3	2.4	2.8	3.5	0.2	3.4	3.7	1.5	0.2	1.3	1.7	1.2	0.4	0.5	1.8
TPSA	108	5.2	104	111	78	4.6	74	81	71	4.1	68	74	71	3.1	69	73	71	4.1	67	73	101	4	98	104	72	3.8	69	76	103	6.5	91	115
Rotatable Bonds	6.5	0.5	6.2	6.9	6	0.4	5.7	6.3	4	0.3	3.8	4.3	5.5	0.4	5.2	5.8	5.6	0.5	5.4	6.1	7	0.3	6.8	7.2	5.3	0.4	5	5.6	3.9	0.3	3.2	4.5
HBDs	2.5	0.2	2.4	2.7	1.7	0.2	1.6	1.9	1.5	0.1	1.4	1.7	1.5	0.1	1.4	1.6	1.4	0.2	1.2	1.5	2.1	0.2	2	2.2	1.7	0.1	1.5	1.8	2.8	0.3	2.3	3.3
HBAs	7	0.3	6.7	7.2	5.6	0.3	5.4	5.8	4.9	0.2	4.8	5.1	4.5	0.2	4.3	4.6	4.8	0.3	4.6	5	7	0.2	6.8	7.2	4.9	0.2	4.7	5.1	6.5	0.4	5.7	7.3
HBDs+HBAs	9.5	0.5	9.1	9.8	7.3	0.4	7	7.7	6.5	0.3	6.2	6.7	6	0.3	5.8	6.2	6.1	0.4	5.8	6.4	9.1	0.3	8.9	9.4	6.6	0.3	6.3	6.9	9.3	0.6	8.1	10
Rings	2.7	0.1	2.6	2.8	2.7	0.1	2.6	2.8	2.1	0.1	2	2.2	2.4	0.1	2.3	2.5	2.2	0.1	2.2	2.3	3.7	0.1	3.6	3.8	2	0.1	2	2.2	1.6	0.1	1.4	1.8
Aromatic Rings	2.6	0.1	2.5	2.7	2.4	0.1	2.3	2.5	2.2	0.1	2.2	2.3	2.4	0.1	2.3	2.5	2.2	0.1	2.1	2.2	3.3	0.1	3.3	3.4	1.8	0.1	1.8	1.9	0.8	0.1	0.7	0.9
Stereocenters	1.2	0.2	1.1	1.4	1.4	0.2	1.2	1.5	1.3	0.2	1.1	1.4	1.5	0.2	1.3	1.7	0.8	0.1	0.7	0.9	1.6	0.2	1.4	1.7	1	0.1	0.9	1.1	5.7	0.4	4.8	6.5
Fsp^3	0.3	0	0.3	0.3	0.4	0	0.3	0.4	0.3	0	0.3	0.3	0.3	0	0.3	0.3	0.3	0	0.3	0.3	0.3	0	0.3	0.3	0.4	0	0.3	0.4	0.6	0	0.6	0.7
Charges	0.5	0.1	0.5	0.6	0.7	0.1	0.7	0.8	0.6	0.1	0.6	0.7	0.4	0.1	0.4	0.4	0.5	0.1	0.4	0.6	0.7	0.1	0.6	0.7	0.7	0.1	0.7	0.8	0.6	0.1	0.4	0.7

Table S1

Mean (M), standard error of the mean (SEM) and 95% CI of the mean (95% lower and upper boundaries are noted LB and UB, respectively, under these conditions, the mean is in the interval between both data values) for the PC computations of all datasets.

Legends to supplementary figures

Figure S1. Caco-2 prediction

Kernel density estimation for human colon carcinoma cells (Caco-2) permeability computed by StarDrop v6.1[1]. Enzymes (light-blue), ion channels (blue), GPCRs (purple), nuclear receptors (yellow), allosteric modulators (brown), iPPIs (orange), OMD (light green) and NPD (dark green).

Figure S2. Metabolic stability prediction – Half-life

Kernel density estimation of metabolic stability (half-life) predicted by StarDrop v6.1[1]. Enzymes (light-blue), ion channels (blue), GPCRs (purple), nuclear receptors (yellow), allosteric modulators (brown), iPPIs (orange), OMD (light green) and NPD (dark green). The molecules with a value above 0.5 are considered as stable.

Figure S3. hERG prediction

Kernel density estimation of hERG pIC50 (15) predicted by StarDrop v6.1[1]. Enzymes (light-blue), ion channels (blue), GPCRs (purple), nuclear receptors (yellow), allosteric modulators (brown), iPPIs (orange), OMD (light green) and NPD (dark green).

Figure S4. Clearance

Kernel density estimation of total clearance (logCL(ml/min/kg)) computed by the pkCSM web-server[2]. Enzymes (light-blue), ion channels (blue), GPCRs (purple), nuclear receptors (yellow), allosteric modulators (brown), iPPIs (orange), OMD (light green) and NPD (dark green).

Figure S5. pLD_{50}

Kernel density estimation of rat LD_{50} predicted by StarDrop v6.1[1]. The negative logarithm of the amount of chemical in mol/kg body weight that causes 50% of rats to die after oral ingestion (Rat oral -log(LD_{50})). Enzymes (light-blue), ion channels (blue), GPCRs (purple), nuclear receptors (yellow), allosteric modulators (brown), iPPIs (orange), OMD (light green) and NPD (dark green).

Figure S6. AMES

AMES mutagenicity predicted by both StarDrop v6.1[1] and the pkCSM web-server[2].

Figure S7. PAINS

Matrix plot of PAINS detection computed by the FAF-Drugs3 server[3]. Frequency (%) is colored as follow: green <0.4, 0.4<yellow<2, 2<orange<4, 4<red<6.2, 6.2<light pink<10, 10<pink<15, 15<dark pink<30, 30<purple<50.

Figure S8. Data set preparations

Visualization of all datasets chemical spaces (filtered dataset in light blue, diversity dataset in red and random dataset in white) obtained with the projection visualization tool of StarDrop 6.1[1], based on path-based fingerprints and Tanimoto similarity.

1 StarDrop v. 6.1 (2016).
2 Pires, D. E., Blundell, T. L. & Ascher, D. B. pkCSM: Predicting Small-Molecule Pharmacokinetic and Toxicity Properties Using Graph-Based Signatures. *J Med Chem* **58**, 4066-4072, doi:10.1021/acs.jmedchem.5b00104 (2015).
3 Lagorce, D., Sperandio, O., Baell, J. B., Miteva, M. A. & Villoutreix, B. O. FAF-Drugs3: a web server for compound property calculation and chemical library design. *Nucleic Acids Res* **43**, W200-207, doi:gkv353 [pii]
10.1093/nar/gkv353 (2015).

Supplementary figures

Figure S1.

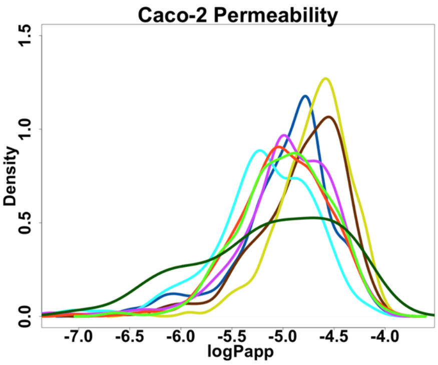

Figure S2.

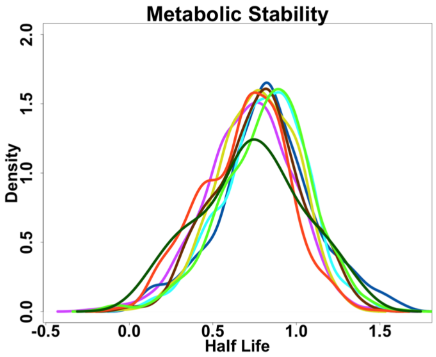

Figure S3.

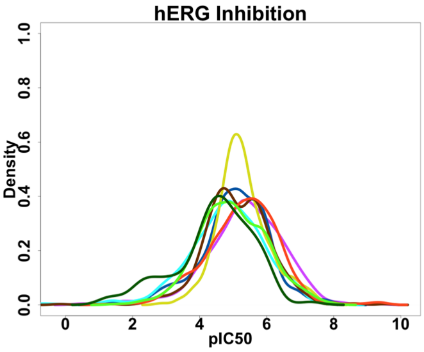

Figure S4.

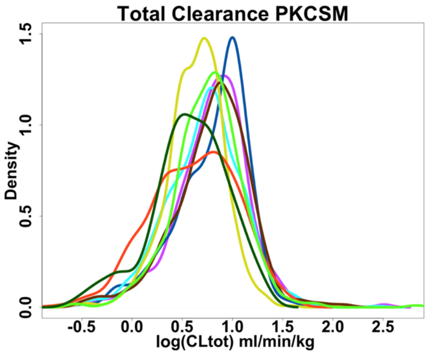

Figure S5.

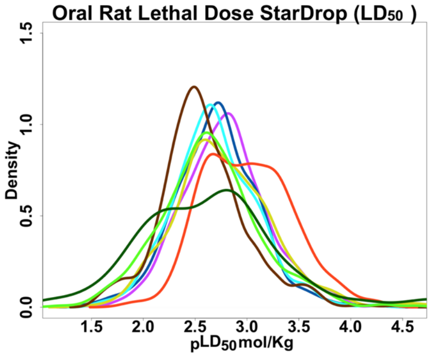

Figure S6.

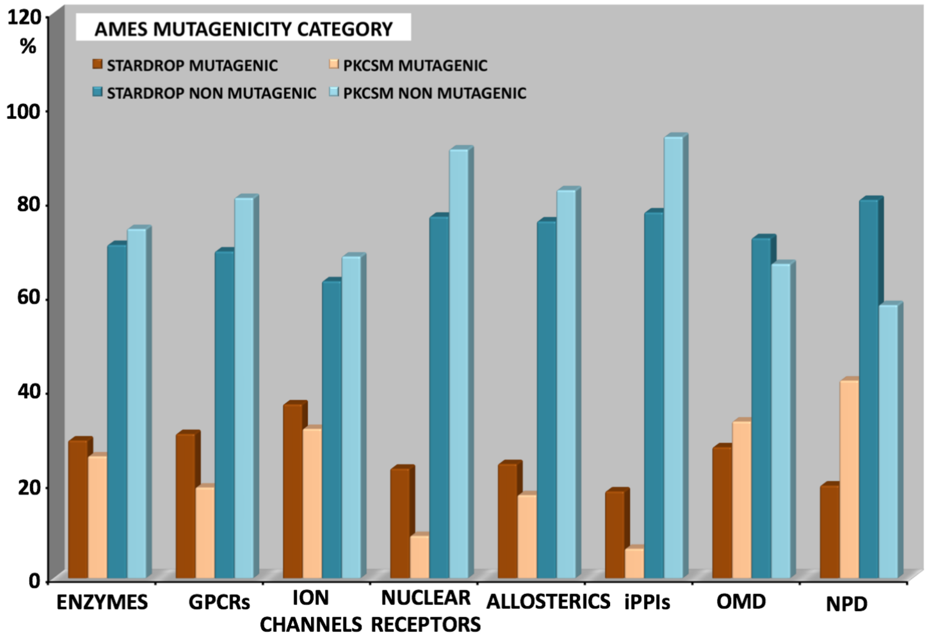

Figure S7.

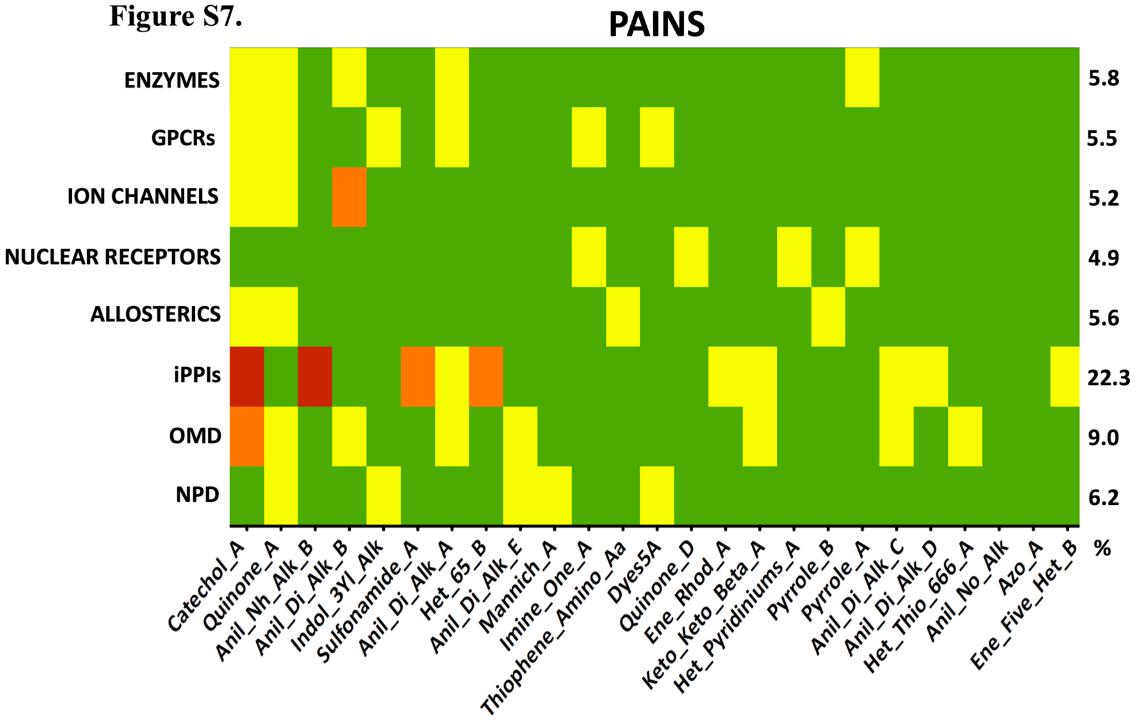

Figure S8

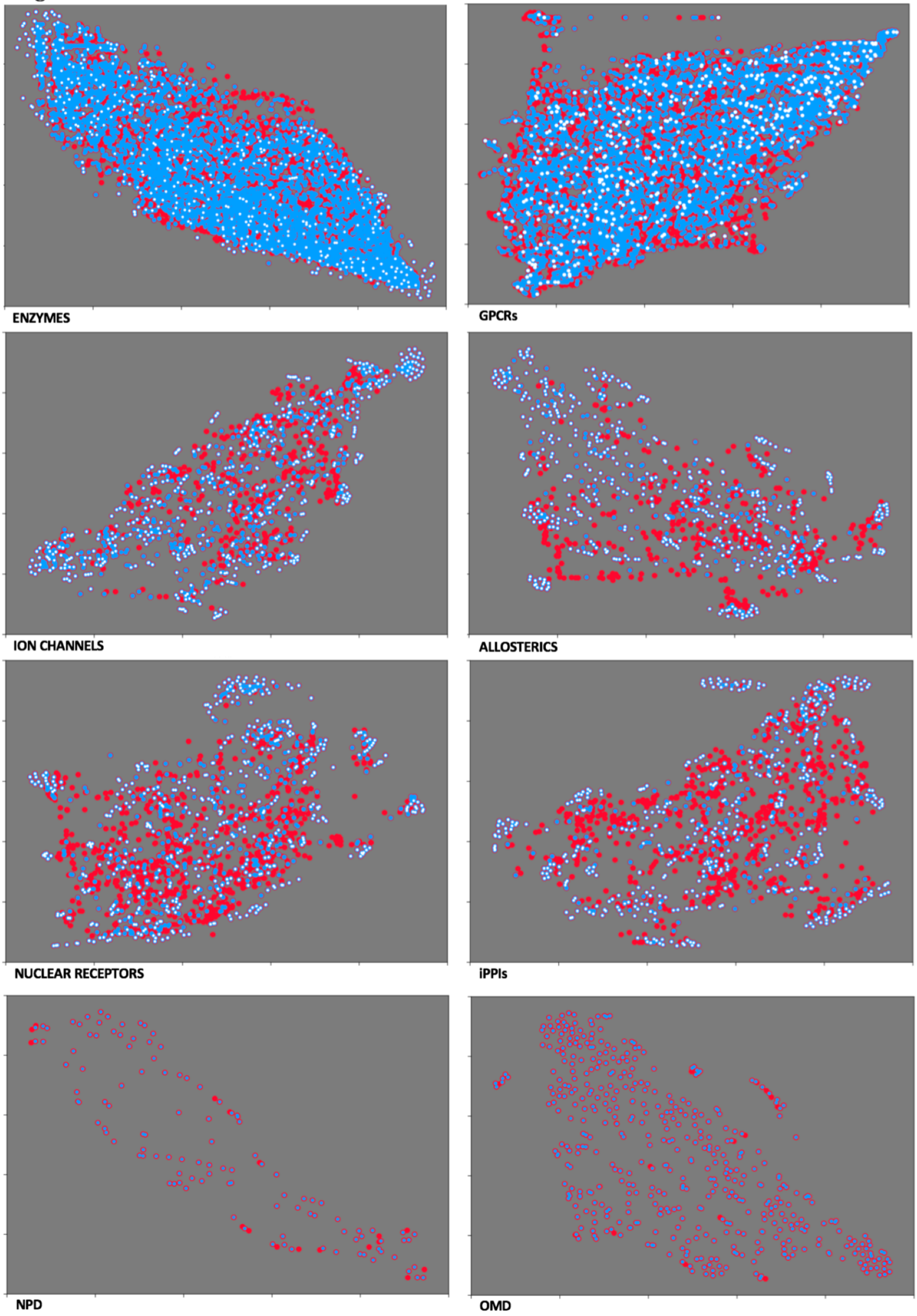
ENZYMES
GPCRs
ION CHANNELS
ALLOSTERICS
NUCLEAR RECEPTORS
iPPIs
NPD
OMD
